# Cases with manifestation of *chemodectoma *diagnosed in dogs in Department of Internal Diseases with Horses, Dogs and Cats Clinic, Veterinary Medicine Faculty, University of Environmental and Life Sciences, Wroclaw, Poland

**DOI:** 10.1186/1751-0147-52-35

**Published:** 2010-05-22

**Authors:** Agnieszka Noszczyk-Nowak, Marcin Nowak, Urszula Paslawska, Wojciech Atamaniuk, Jozef Nicpon

**Affiliations:** 1 Department of Internal Diseases with Horses, Dogs and Cats Clinic, Veterinary Medicine Faculty, University of Environmental and Life Sciences Wroclaw 50-366, Poland; 2 Department of Pathological Anatomy Veterinary Medicine Faculty, University of Environmental and Life Sciences Wroclaw 50-366, Poland; 3 Department and Clinic of Surgery, Veterinary Medicine Faculty, University of Environmental and Life Sciences Wroclaw 50-366, Poland

## Abstract

In the period of 3 years, 9 tumours of *chemodectoma *were supravitally diagnosed and histopathologically verified in dogs. In this period 15 351 dogs were admitted to the Clinic of Dogs and Cats and 2 145 dogs were examined in the cardiological outpatient clinic for dogs. This tumour is located in a typical place - at the base of the heart. Most frequently the tumour manifested in older boxers. Only in one case such a tumour was diagnosed in another breed of dogs. The tumours ranged in size between 3 and 16 cm in diameter. The principal sign accompanying tumours of cardiac base involved dyspnoea but in 3 cases the tumours yielded no clinical signs. All the diagnoses were additionally verified using immunohistochemical examination. We used antibodies to chromogranin A (clone DAK-A3 1:100), synaptophysin (clone SY38 1:20) and neuron-specific enolase (clone BBS/NC/VI-H14 1:150). An immunohistochemical examination is vital for the diagnosis since it allows to differentiate histologically distinct types of neoplasia which may locate in the same site and may manifest a similar histological pattern.

## Introduction

*Chemodectomas *s. *paragangliomas *represent tumours derived from chemoreceptor cells originating most frequently from aortic body or carotid glomus [1]. The site of their origin may involve also the tympanic cavity and inferior vagal ganglion. In animals the tumours manifest most frequently in the form of a single tumour located at the base of the heart. Less frequently they form accumulation of small tumours. Occasionally, they infiltrate myocardium [2]. *Chemodectoma *may manifest traits of a malignant tumour or of a benign tumour [1,3]. It should be added that in most cases *chemodectoma *metastases are infrequently encountered [4]. Tumour neuroendocrine cells (type I cells) may produce and secrete catecholamines and serotonin [5,6]. The authors described secretory granules in *chemodectoma *tumour cells, even if DJ Meutena is his book Tumours claims that in animals chemodectomas do not produce catecholamins [7]. Catecholamines may induce disturbances in the cardiac rhythm, not infrequently associated with *chemodectoma *[8]. Diagnosis and treatment of cardiac tumours continue to be difficult. The diagnosis takes advantage of the most modern imaging techniques, i.e., ultrasonography, radiography and magnetic resonance [3,9]. Their accurate diagnosis, however, apart from standard staining with hematoxylin and eosin requires application of immunohistochemistry with use of antibodies specific for chromogranin A, synaptophysin and neuron-specific enolase [1,10-13].

The present study aimed at analysis of manifestation and characteristics of *chemodectoma *type cardiac base tumours.

## Materials and methods

The studies were performed on 9 dogs, patients of the Department of Internal Diseases with Horses, Dogs and Cats Clinic, and the Department and Clinic of Surgery, Veterinary Medicine Faculty, University of Environmental and Life Sciences. In 6 dogs the reason for the consultation of veterinary doctor was a pronounced dyspnoea. In 2 dogs the tumour of the cardiac base was diagnosed during cardiological examination, performed before surgical procedure. In one dog the tumour was detected during radiological examination of the chest, performed due to suspected fracture of the ribs following a traffic accident. In all the dogs morphology and biochemistry of venous blood was carried out, gasometric tests on arterial blood, echocardiography of the heart, chest X-ray and ECG examination were performed. All the dogs were subjected to euthanasia in the period ranging from 3 days to 18 months following the diagnosis of the cardiac base tumour. During autopsy, samples of the tumour, myocardium of the right and the left atrium, the right and the left ventricle, intraventricular septum, lungs, liver and kidneys were taken and fixed in a buffered 7% solution of formalin. Staining with hematoxylin and eosin was performed and, then, immunohistochemical studies were conducted with the use of antibodies to chromogranin A, synaptophysin and neuron-specific enolase (NSE). The sections were mounted on Superfrost slides (Menzel Gläser, Germany), dewaxed with xylene and gradually rehydrated. Activity of endogenous peroxidase was inhibited by 5 min exposure to 3% H_2_O_2_. Detection of chromogranin A, synaptophysin and neuron-specific enolase antigen expression was preceded by 15 min exposure of the sections in a microwave oven to a boiling Antigen Retrieval Solution (DakoCytomation, Denmark) at 250 W. For demonstration of chromogranin A, synaptophysin and neuron-specific enolase antigen expression in the paraffin sections, antibodies were used in the following concentrations: clone DAK-A3 (1:100) (DakoCytomation, Denmark); clone SY38 (1:20) (DakoCytomation, Denmark); clone BBS/NC/VI-H14 (1:150) (DakoCytomation, Denmark). The antibodies were diluted in the Antibody Diluent, Background Reducing solution (DakoCytomation, Denmark). The sections were incubated with an antibody for 1 h at room temperature. Subsequently, incubations were performed with biotinylated antibodies (15 min, room temperature) and with streptavidin-biotinylated peroxidase complex (15 min, room temperature) (LSAB2, HRP, DakoCytomation, Denmark). DAB (DakoCytomation, Denmark) was used as a chromogen (7 min, room temperature). All the sections were counterstained with Meyer's hematoxylin. In every case controls were included, in which specific antibody was substituted by the Primary Negative Control (DakoCytomation, Denmark).

Depending on the value of proliferation index, cell morphology, possible infiltration into interior of vascular wall or myocardium as well as presence or absence of metastases the tumours could be categorized to three grades of malignancy: I (benign) - low proliferation index (up to 2 mitotic figures per 1000 cells), relatively uniform cells, no infiltration of the neighbourhood and no metastases, II (malignant) - low to average proliferation index (2 to 10 mitotic figures), less uniform cells, presence of tumour regions with infiltrative growth, possible presence of metastases, III (malignant) - high proliferative index (more than 10 mitotic figures), evident cellular pleomorphism, infiltrative growth, presence of metastases [1]. Macroscopically, benign tumours demonstrate absence of necrotic foci or haemorrhages. In most of benign hyperplasias cells were of a similar size and shape and hyperchromasia was infrequently encountered.

## Results

**Figure 1:**
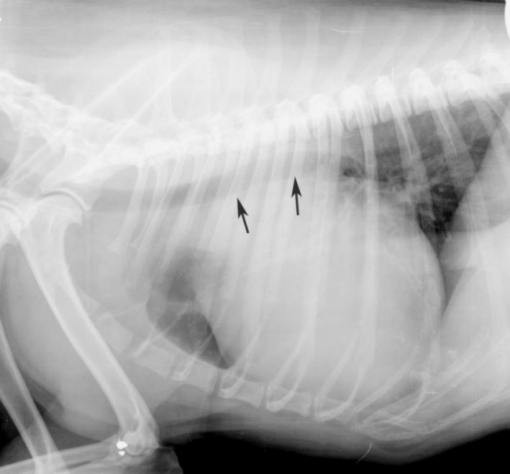
**Radiogram of chest in right-left lateral projection**. Presence of the large tumour in mediastinum elevated trachea in dorsal direction (black arrows). Outline of the tumour remains invisible since its shadow overlaps with that of the other mediastinal organs, including the heart. Case no 2.

**Figure 2:**
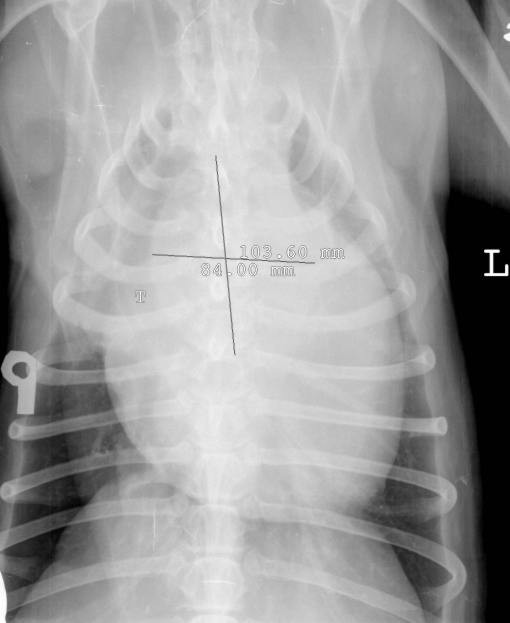
**Radiogram of chest in ventro-dorsal projection**. The ventro-dorsal projection of the chest visualizes well the left and the right edge of the tumour. The widest dimension of the tumour amounts to around 8.4 cm. Note the transplaced to the right trachea (T). The approximate length amounts to 10.3 cm. Case no 2.

**Figure 3:**
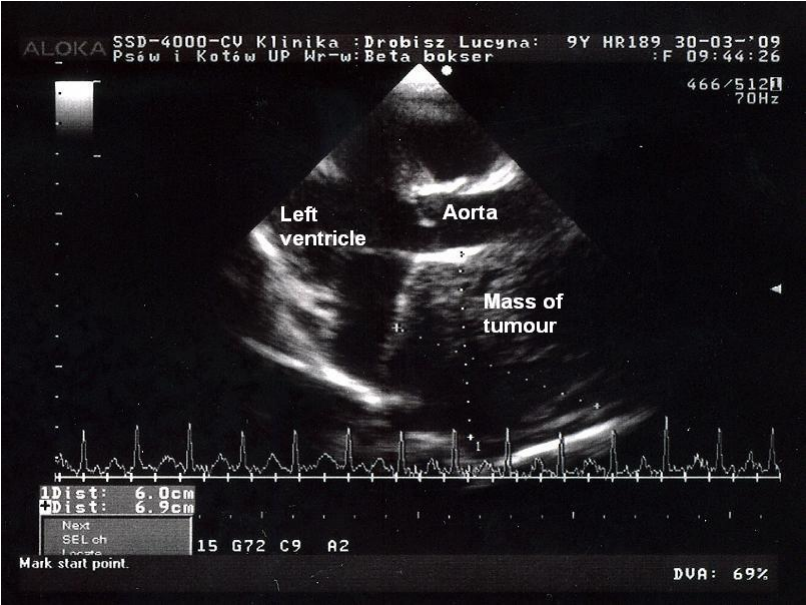
**Echocardiography image in apical projection**. Tumour of heart base seen near the aorta. Case no 4.

**Figure 4:**
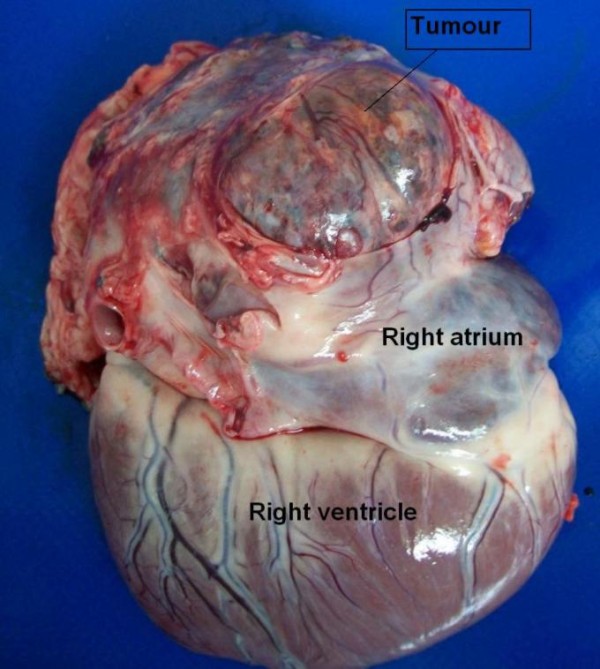
**Autopsy**. Tumour of heart base. Case no 1.

**Figure 5:**
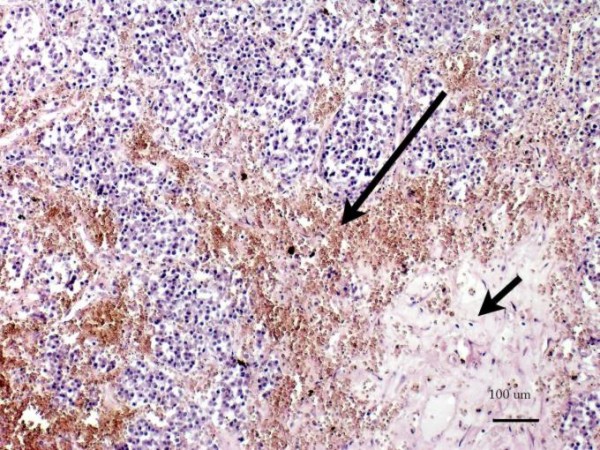
**Micrographs**. It showing dispersed foci of haemorrhage (long arrow) and extensive region of necrosis in centre of the tumour (short arrow) in malignant forms of the tumour.

**Figure 6:**
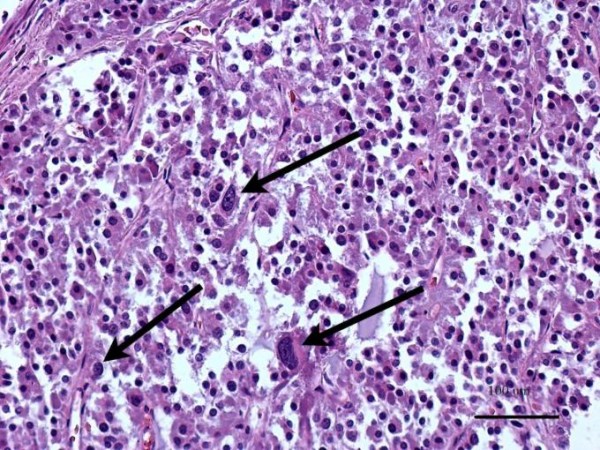
**Micrographs**. It showing extensive polymorphism of cell nuclei (arrows) and their hyperchromasia in malignant cells of the tumour.

Dogs in which the tumour of the cardiac base was diagnosed, were between 8 and 13 years old (mean 9.7 1.8) (Table 1). The group included 5 male and 4 female dogs. Among the male dogs one was subjected to castration 3 years before diagnosis of the tumour of the cardiac base. Among the female dogs two were subjected to ovario-hysterectomy 5 to 7 years before diagnosis of the cardiac base tumour. Among the studied dogs, boxers prevailed (8 dogs), only one represented another breed (a dachshund). The tumours of the cardiac base in all the cases could be detected by chest X-ray (Figs. 1 and 2), in 6 cases they were also detected by echocardiography (Fig. 3). Blood morphology and biochemistry (WBC, RBC, PLT, Hb, Ht, urea, creatinine, ALT, AST, FA, K^+^, Na^+^, Ca^2+^) detected no abnormalities although deviations were noted by gasometry (Table 2). Among the signs, dyspnoea and fatigability prevailed. In 5 out of the 9 dogs a hydrothorax and in 2 a hydropericardium were noted. In all 5 dogs with hydrothorax and in 2 dogs with hydropericardium the fluid contained no atypical cells. In 5 out of the 9 dogs resting ECG examination detected numerous precocious, single and paired ventricular beats. In one dog an attack of ventricular tachycardia was detected. The tumours ranged from 3 cm to 16 cm in diameter (Fig. 4), they manifested a firm consistency, mosaic colour and each was surrounded by a connective tissue capsule. All the tumours manifested a junction with the aortic arch, noted even in the ultrasonographic examination (Fig. 3). Tumours exceeding 8 cm in diameter contained foci of necrosis and haemorrhages (Fig. 5). In a single case the tumour contained several cavities. In all the cases the tumour was verified to represent *chemodectoma*. In 4 cases neoplastic cells infiltrated a blood vessel and/or the cardiac atrium. In none of the cases could metastases to other inner organs be detected. In all 9 cases of the cardiac base tumours histopathological examination detected neoplastic hyperplasia of *chemodectoma*, in its either benign (5 cases) or malignant (4 cases) form (Table 1). The tumour cells formed numerous foci of a variable size, separated by trabeculae of connective tissue, well supplied with blood. Most tumour cells manifested a polygonal outline, a spherical or oval cell nucleus and individual nucleoli. In contrast to benign forms, the cases of malignant forms demonstrated locally numerous, frequently abnormal mitotic figures. The cytoplasm was finely granular with numerous vacuoles of various shape and size. The characteristic trait of malignant forms was an extensive polymorphism of cell nuclei and their hyperchromasia (Fig. 6). Moreover, a rich blood supply was evident, with numerous muscular arterioles, thin-walled veins and a network of fine capillaries within the connective tissue septa. In some regions of the tumours cells formed a radial pattern around blood vessels. The microscope examination of the malignant forms demonstrated also dispersed areas of haemorrhages and an extensive necrotic region in the tumour centre (Fig. 5). The peripheral part of the tumours contained fine, mainly lymphohistiocytic inflammatory infiltrates.

**Table 1:** Tumour size and its manifestation in laboratory tests

Case No	Breed	Sex	Age (years)	Tumour diameter	Radiological examination	USG	Grade of malignancy	ECG
1	boxer	M	13	16.0 cm	detectable	detectable	III	Numerous, individual or paired premature ventricular beats

2	boxer	F	8	9.0 cm	detectable	detectable	II	Numerous, individual premature ventricular beats

3	boxer	F	11.5	4.0 cm	detectable	not detectable	Benign	Ventricular tachycardia

4	boxer	M	11	7.0 cm	detectable	detectable	II	Numerous, individual premature ventricular beats

5	boxer	M	9	4.5 cm	detectable	not detectable	II	Numerous, individual premature ventricular beats

6	boxer	M	10	11.0 cm	detectable	detectable	Benign	Numerous, individual or paired premature ventricular beats

7	boxer	F	8.5	3 cm	detectable	not detectable	Benign	No arrhythmia

8	boxer	M	8	5 cm	detectable	detectable	Benign	No arrhythmia

9	dachshund	F	8	5 cm,	detectable	detectable	Benign	No arrhythmia

**Table 2:** Results of blood tests

Case No	1	2	3	4	5	6	7	8	9	Standard
Breed	boxer	boxer	boxer	boxer	boxer	boxer	boxer	boxer	dachshund	

Sex	M	F	F	M	M	M	F	M	F	

age [years]	13	8	11.5	11	9	10	8.5	8.0	8	

pH	7.43	7.37	7.38	7.36	7.37	7.36	7.39	7.36	7.37	7.35-7.45

pCO2 [mmHg]	45	50	48	49	48	49	51	51	49	35-45

pO2 [mmHg]	62	46	62	40	46	48	50	42	46	85-100

BE [mmol/l]	-2.1	-2.8	-2.6	-2,8	-2.1	-2.4	-2.5	-2.1	-2.2	2 - (-2.0)

**Figure 7:**
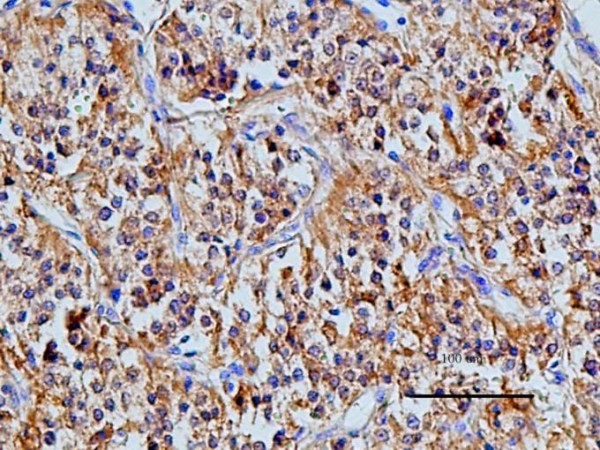
**Expression of chromogranin A in cytoplasm of chemodectoma cells**.

**Figure 8:**
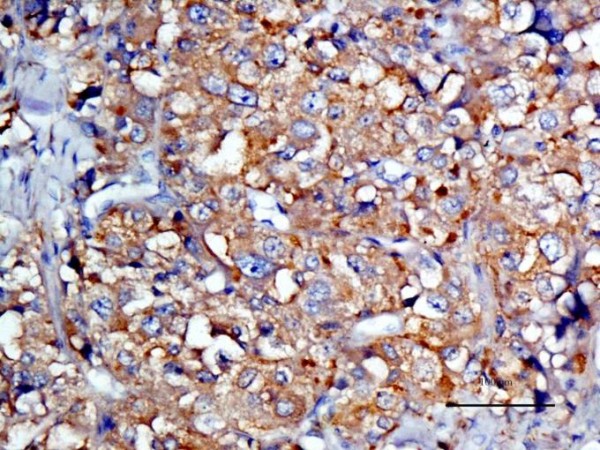
**Expression of synaptophysin in cytoplasm of chemodectoma cells**.

**Figure 9:**
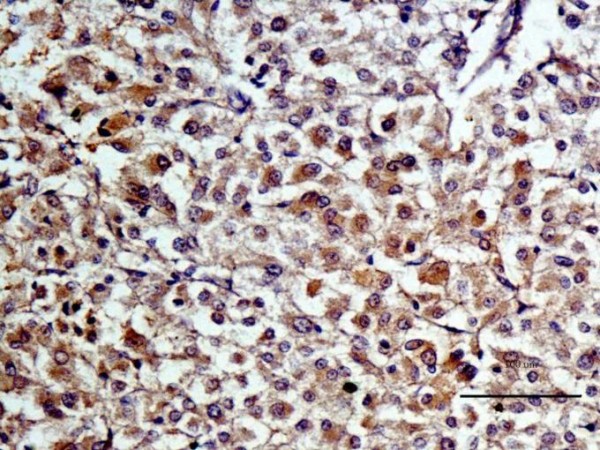
**Expression of neuron-specific enolase (NSE) in cytoplasm of chemodectoma cells**.

In order to verify the results of the standard histopathological examination with hematoxylin-eosin staining, immunohistochemistry was applied with use of a panel of antibodies, specific for tumour cells of neuronal or neuroendocrine origin [12-14]. All the antibodies yielded a positive reaction with a fine granular, brownish reaction, localized mainly in the cytoplasm of the tumour cells (Fig. 7, 8 and 9), which confirmed the preliminary diagnosis. Negative control demonstrated no colour reaction, thus confirming that the procedure was properly conducted.

## Discussion

Tumours of the cardiac base usually develop in older animals [1,15]. In the studied group of dogs the main age was to 9.8 ± 1.8 years. A breed particularly predisposed to such tumours involves boxers, which in our group accounted for 9/10 (89%) of the affected dogs [9]. In the years 2006-2008, 167 dogs of the boxer breed were examined. In that group a cardiac disease with arrhythmias was diagnosed in 71 boxers and a *chemodectoma *was confirmed in 9 dogs, accounting for 5.4% of the examined population and for 12.7% of the examined dogs with cardiac disease. A single dog with the diagnosed tumour of the cardiac base of *chemodectoma *has represented another breed (a dachshund). In the years 2006-2008, 1472 dogs of the dachshund breed were examined in our clinic, in that group 617 dachschunds manifested cardiac disease (571 dogs suffered from heart failure, 46 manifested arrhythmias). In this time, among other animals, 896 German Sheppard Dogs, 267 Dobermans, 594 Yorkshire Terriers were consulted in the clinic. They are the most popular breeds in Poland. Manifestation of tumours of this type in boxers may be linked to the type of structure in the upper respiratory tract (brachycephalic airway syndrome -BAS). The primary features of BAS include stenotic nares, an elongated soft palate, and distortion of the pharyngeal soft tissues due to restral shortening of the skull. These abnormalities are seen in brachycephalic breeds and they increase resistance to airflow in the upper respiratory tract. The increased negative pressure required during inspiration may result in secondary changes including laryngeal collapse. These abnormalities cause the chronic hypoxia and are suspected to provide a cause of hyperplasia and, finally, neoplasia of chemoreceptor cells [1,6]. In 1969, Arias-Stella reported enlargement of the carotid bodies in high-altitude dwellers in the Peruvian Andes. The carotid bodies of guinea pigs, rabbits and dogs which had been born and lived all their lives in Peru (altitude of 4330 meters) were found to be enlarged. Climate can influence the development of neoplasms. Peruvian adults born and living at high altitude in the Andes have high incidence of *chemodectoma*. It is related to chronic hypoxia. In cattle living at high altitude Ariss-Stella and Valcarcel observed carotid bodies with extreme chief cell hyperplasia and the *chemodectoma *was present in 40% of the animals [5]. The assumed relationship seems to be confirmed by the results of gasometric tests on arterial blood, which have clearly demonstrated a lower partial pressure of oxygen, increased partial pressure of carbon dioxide, decreased base excess (BE) and normal pH. It is a pattern typical of compensated respiratory acidosis. The respiratory acidosis may develop in boxers due to their structural type of the respiratory tract, representing part of the so-called brachycephalic syndrome. Large size tumours of the cardiac base obviously may induce respiratory disturbances leading to acidosis but in our study respiratory acidosis developed also in dogs with small tumours (3-4.5 cm). Such small tumours are not able to compress bronchi and cannot mechanically interfere with gas exchange. These observations might indicate that respiratory acidosis represents the primary disturbance and that it might predispose dogs of the boxer breed to development of *chemodectoma*. In the dog of the dachshund breed a probable cause of poor gas exchange involved nasal polyps. It should be added that in other brachycephalic breeds, such as, e.g., Pekinese the tumour does not develop more frequently than in general population of dogs but an increased incidence of the tumour was described in Boston terriers and, thus, a family predisposition to development of *chemodectoma *is possible [6]. Among the signs, dyspnoea dominated, affecting 6/9 (67%) of the dogs. It resulted from hydrothorax and pressure exerted by the large tumours on the main bronchi. In one of the dogs hydrothorax was accompanied by hydropericardium. In the other case, hydropericardium represented the only sign of the cardiac base tumour. The heart base tumours could always be detected in the radiological examination of the chest, in the lateral and dorso-ventral projection. The ultrasonographic examination of the heart detected only the tumours which exceeded 4.5 cm in diameter. In view of the obtained results, the radiological examination has proven more sensitive in detection of cardiac base tumours than the ultrasonographic examinations. Differential diagnoses should comprise metastasis, mediastinal abscess, lymph node inflammation in mediastinum, lymphoma. Metastases were excluded in post-mortem examination, mediastinal abscess, inflammation of lymph nodes and lymphoma were excluded by morphological and biochemical examination of blood. Type I (neuroendocrine) cells of *chemodectoma *contain secretory granules with catecholamines which may induce ventricular arrhythmias. In the studied dogs with *chemodectoma *numerous premature ventricular beats were detected. One dog manifested attacks of ventricular tachycardia, which disappeared following intravenous administration of a beta-blocker but did not vanish following administration of lidocaine, which is the drug of choice. This might be interpreted as consistent with the potential for production of catecholamines by chemodectoma tumours. The described location of the tumour might predispose to supraventricular arrhythmias which, however, were not recorded in the examined dogs. Blumcke et al examined reaction of carotid bodies in rats to acute and severe oxygen deficiency. They detected the almost total discharge of catecholamines from type I cells after 20 minutes of extreme hypoxia, which was confirmed by fluorescence microscopy. Mitchell postulated that catecholamines in the type I cells may function as transmitters in an efferent neuronal pathway. *Chemodectoma *represents a tumour which usually does not metastasize. Nevertheless, some cases of metastases were described - to lungs, liver, myocardium and even to brain and bones [1]. In all the cases the tumour was verified to represent chemodectoma; five benign and four malignant cases. In malignant cases neoplastic cells infiltrated a blood vessel and/or cardiac atrium with no metastases to other organs.

Out of all the applied markers, used to detect neoplastic cells of neuronal or neuroendocrine origin, the highest significance was linked to chromogranin A. The protein, as a protein prohormone, represents a marker of neuroendocrine differentiation and the directed against it antibodies are used to identify cells and tumours of neuroendocrine origin. Expression of NSE and that of synaptophysine manifested a similar level in the cells of both benign and malignant tumours. On the other hand, in the case of chromogranin A we, similarly to Aresu et al. [1], have found that expression of the marker decreases with increasing malignancy of the tumour.

Our observations have shown that all the applied antibodies are useful in differential diagnosis of neoplastic processes located at the cardiac base but only chromogranin A may help in evaluation of tumour malignancy.

Summing up, *chemodectoma *develops in dogs of the boxer breed significantly more frequently than in the general population of dogs. As demonstrated by our observations, the sensitive examination, which allows to detect them, involves the radiological examination of the chest. The examination of the fluid drained from the pleural or pericardial cavity is useless in the diagnosis of *chemodectoma *since tumour cells are not des quamated to the fluid. Gasometric blood tests may be helpful in selecting boxers with the risk of *chemodectoma *development. The diagnosis requires confirmation by the histopathological examination.

## Competing interests

The authors declare that they have no competing interests.

## Authors' contributions

ANN carried out ECG and echocardiographic examinations, blood collection and interpreted the tests, drafted the manuscript. UP carried out echocardiographic examinations. MN performed the histopathological examinations and drafted the manuscript. WA carried out X-ray examinations. JN interpreted blood analysis and drafted the manuscript. All authors read and approved the final manuscript.
